# The Possible Role of Glucose-6-Phosphate Dehydrogenase in the SARS-CoV-2 Infection

**DOI:** 10.3390/cells11131982

**Published:** 2022-06-21

**Authors:** Israel Pérez-Torres, María Elena Soto, Verónica Guarner-Lans, Linaloe Manzano-Pech, Elizabeth Soria-Castro

**Affiliations:** 1 Department of Cardiovascular Biomedicine, Instituto Nacional de Cardiología “Ignacio Chávez”, Juan Badiano 1, Sección XVI, Tlalpan, México City 14080, Mexico; loe_mana@hotmail.com; 2 Department of Immunology, Instituto Nacional de Cardiología “Ignacio Chávez”, Juan Badiano 1, Sección XVI, Tlalpan, México City 14080, Mexico; elena.soto@cardiologia.org.mx; 3 Department of Physiology, Instituto Nacional de Cardiología “Ignacio Chávez”, Juan Badiano 1, Sección XVI, Tlalpan, México City 14080, Mexico; veronica.guarner@cardiologia.org.mx

**Keywords:** Glucose-6-phosphate dehydrogenase, SARS-CoV-2, COVID-19, redox homeostasis, Warburg effect, pentose phosphate pathway

## Abstract

Glucose-6-phosphate dehydrogenase (G6PD) is the second rate-limiting enzyme of the pentose phosphate pathway. This enzyme is present in the cytoplasm of all mammalian cells, and its activity is essential for an adequate functioning of the antioxidant system and for the response of innate immunity. It is responsible for the production of nicotinamide adenine dinucleotide phosphate (NADPH), the first redox equivalent, in the pentose phosphate pathway. Viral infections such as SARS-CoV-2 may induce the Warburg effect with an increase in anaerobic glycolysis and production of lactate. This condition ensures the success of viral replication and production of the virion. Therefore, the activity of G6PD may be increased in COVID-19 patients raising the level of the NADPH, which is needed for the enzymatic and non-enzymatic antioxidant systems that counteract the oxidative stress caused by the cytokine storm. G6PD deficiency affects approximately 350–400 million people worldwide; therefore, it is one of the most prevalent diseases related to enzymatic deficiency worldwide. In G6PD-deficient patients exposed to SARS-CoV-2, the amount of NADPH is reduced, increasing the susceptibility for viral infection. There is loss of the redox homeostasis in them, resulting in severe pneumonia and fatal outcomes.

## 1. Introduction

### Glucose-6-Phosphate Dehydrogenase

Glucose-6-phosphate dehydrogenase (G6PD) is a rate-limiting enzyme in the pentose phosphate pathway (PPP). It is responsible for the generation of nicotinamide adenine dinucleotide phosphate (NADPH) [1], which is involved as a cofactor in the homeostasis of glutathione (GSH), and other enzymes such as glutathione reductase, the glutathione peroxidases family, and the thioredoxin family. It regulates the activity of many other enzymes including the fatty acid synthase, super oxide dismutase isoforms, catalase, NADPH oxidases, nitric oxide synthase isoforms, and methemoglobin reductase [2]. It is also involved in the mitochondrial transport chain, cholesterol synthesis, fatty acid synthesis [3], and steroid hormone production [4].

G6PD is present in the cytoplasm of all mammalian cells, and it is encoded by a gene located in the terminal region of the long arm of the X chromosome (Xq28). It is distant less than 2 centi-Morgans from the gene that encodes for factor VIII. The hereditary condition linked to the X chromosome in men determines its homozygous character in them [5]. Its gene consists of 12 introns and 13 exons, and it encodes for the primary peptide, which folds to form a monomer of 515 amino acids. Two monomers interact to form dimers that subsequently form NADP^+^-dependent tetramers [6] Catalytic activity only begins when an equilibrium state between the dimeric and the tetrameric forms is established [5]. G6PD activity is essential for the adequate functioning of the antioxidant system and for innate immunity, which are needed to counteract COVID-19 [7].

In this review article, we summarize information on whether there is an increase or a decrease in the expression and/or activity of G6PD in COVID-19 patients and provide preliminary images supporting that there is an elevation in its expression. We discuss the effects of increases or decreases in this enzyme on the risk of getting infected by SARS-CoV-2 and on the possible outcome of the disease.

## 2. Changes in Metabolic Pathways Induced by Viral Infections

Viruses are obligatory intracellular parasites that depend on the metabolic machinery of the host cell to supply the necessary energy and molecular building blocks needed for successful replication. During replication, viruses modulate the host cell metabolic homeostasis to boost activities such as glycolysis, the PPP pathway, and fatty acid metabolism to fulfill the viral energy requirements. Non-structural and structural proteins and lipids from the membrane bilayer are needed for the synthesis of the viral genome and capsule. The mitochondrial function of the host is also hijacked by different viruses including the SARS-CoV-2, and they shift it from aerobic to anaerobic [8]. Thus, the pyruvate produced from glucose during the process of glycolysis is reduced to lactate. Glucose levels in the cytosol are increased and there is a reduction of the production of adenosine-5-triphosphate (ATP) [9].

Large quantities of ATP are needed for viral replication and, therefore, there is a depletion of the ATP concentration. In this situation, gluconeogenesis does not use lactate, and this metabolite accumulates in the blood, promoting tissue acidification and exhausting immune cells [9]. At the same time, this condition contributes to increase lactate dehydrogenase that is the enzyme that catalyzes the conversion of pyruvate to lactate in anaerobic conditions [10]. Moreover, lactate levels elevate the liver dependency on glycolysis, which leads to an elevation of glucose in the blood. Viral replication is promoted in this scenario, since the virus needs large amounts of energy for biosynthesis [11].

Hyperglycemia constitutes a high risk for complicated COVID-19, particularly in people without previous diabetes or with diabetes discovered at hospitalization [12]. Risk for mortality related to COVID-19 is also increased in patients with diabetes and poor glycemic control before infection is observed [13]. In addition, high glucose concentrations result in an elevated activity of the PPP pathway [14]. Over-activation of the PPP and the hyperglycemic state are crucial mechanism in the inflammatory process where high pro-inflammatory cytokines are produced [8]. In this condition, there is an excess formation of free radicals and there is loss of systemic redox homeostasis, which leads to an exaggerated response of the positive feedback process in the axis established between the cytokine storm-inflammation and oxidative stress. This could result in fatal outcomes for patients with severe pneumonia associated to the infection by SARS-CoV-2. This is further aggravated in patients with other pathologies such as metabolic syndrome [7].

The increase in the activity of the PPP is the result the Warburg effect that certain viral infections induce to ensure the success of the viral replication process and virion production [15]. The Warburg effect is described as the process by which the cells obtain energy by anaerobic glycolysis through lactic acid fermentation mainly in the cytosol. This is in contrast to its production by the normal aerobic pyruvate oxidation in the mitochondria [16]. The Warburg effect leads to severe hypoxia, thrombosis, pulmonary arterial hypertension, and acute respiratory distress syndrome in SARS-CoV-2 infection [16]. This effect is also present in infections by the Zika virus, human cytomegalovirus, and the coronavirus responsible of transmissible gastroenteritis, in which the membrane glucose transporters-1, -2, and -4 and the apical transporters Na^+^-1-dependent glucose transporters are disturbed [17,18].

The Warburg effect leads to an increase in the activity of hexokinase (HK), which is the first rate-limiting enzyme of glycolysis through the PPP. The function of HK is to convert glucose to glucose-6-phosphate (G6P), which is subsequently oxidized by G6PD for the formation of the first NADPH molecule [18,19]. In mammalian cells, there are four HK isoforms: HK-1, HK-2, HK-3, and HK-4 [19]. The activity of HK-2 especially promotes the increase of the flux of glucose due to the upregulation of downstream glycolytic enzymes [20]. Moreover, HK-2 is upregulated in several viral infections, such as those produced by the serotype 1 Dengue virus and the hepatitis C virus [21], the Rous sarcoma virus, the alphavirus Mayaro, the hepatitis B virus (HBV), kaposi’s sarcoma-associated herpesvirus, and the human cytomegalovirus [17]. It has also been described that human immunodeficiency virus type 1 (HIV-1) can induces HK-1 over-expression in human monocytes and macrophages [19]. However, the activity of HK may decrease after 24 h of post-viral infection [22].

As a result of the PPP upregulation, the ribose needed for viral biosynthesis of nucleotides and nucleic acids is generated [23]. Many viruses including the influenza virus, hepatitis C virus, cucumber mosaic virus, and HIV-1 are able to increase the PPP flux to stimulate ribose-5-phosphate generation for nucleic acid and nucleotide synthesis [24]. In this sense, the increase in HK results in more G6P that favors an elevation of the activity of the G6PD.

## 3. G6PD and Viral Infections

### 3.1. Effects of Increased Activity of G6PD

G6PD is increased in different viral infections including the infectious pancreatic necrosis virus, the influenza virus, HIV, and cucumber mosaic virus, white spot syndrome virus, and infectious bursal disease virus [25,26,27]. In the spleen and liver of mice infected with Friend virus, the G6PD was slightly increased [28]. Even in viral plant infections, the activity of G6PD is increased. For example, in plum pox virus infection, there is an increase of the G6PD [29]. The above suggests that elevations in G6PD activity in animal and plant cells after a viral infection is associated with the viral cycle and with an increase in the radical oxygen species (ROS) [30]. ROS such as H_2_O_2_ are produced by cells, such as macrophages and neutrophils, as a mechanism to destroy viral particles as part of a defense mechanism that halts the viral replication and its harmful effects on tissues. However, overproduction of ROS or their incapacity to discriminate between exogenous pathogens, such as SARS-CoV-2 and endogenous host tissue, leads to tissue damage.

There is an association between an excess of G6PD expression and an elevated activity of the inducible nitric oxide synthase in the airway epithelial cells during acute lung injury [31]. In diabetes, there is as association between increased activity of G6PD and endothelial and vascular dysfunction and elevated levels of NADPH. There is also an elevation in myocardial G6PD expression and activity in heart failure corrected by induced pacing [32]. In addition, there was an increase in the expression of G6PD, through the Nrf2 pathway in the HBV infection in hepatocytes [33]. There was also over-expression of the G6PD in patients with papillomavirus infection in human cervical cancer [34]. A pilot study in patients with COVID-19 showed a significant increase in the levels of G6PD activity in red blood cells and it was concluded that the increase in G6PD was a compensatory mechanism against the viral infection [35].

**Figure 1:**
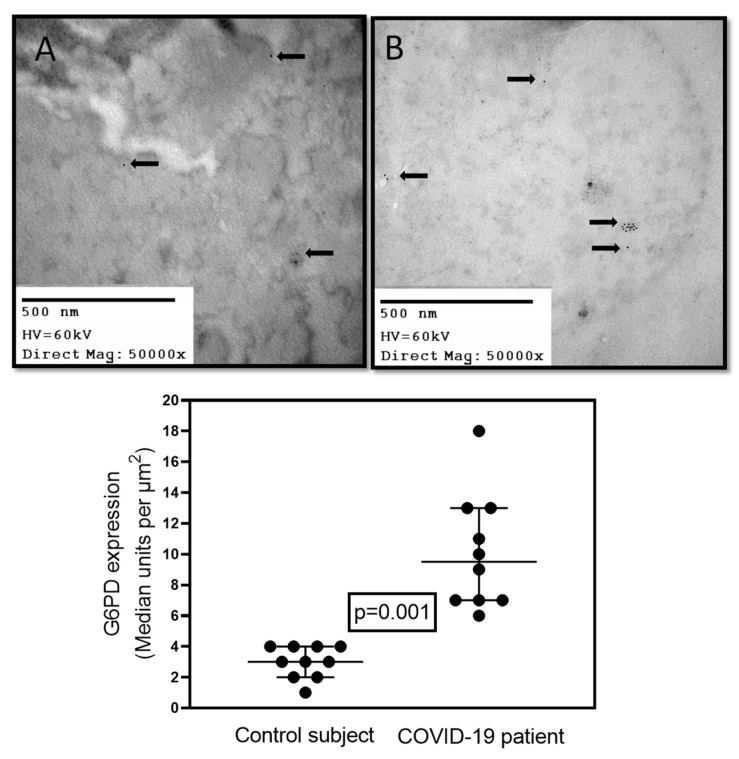
Representative immune-electron micrograph of lung. (**A**) Postmortem sample from a biopsy from a 59-year-old female control subject with hypertension, obesity, type 2 diabetes, and pneumonia. (**B**) Postmortem sample from a 68-year-old female patient with COVID-19 that had associated comorbidities of ischemic heart disease, type II diabetes, morbid obesity, and hypertension without G6PD deficiency. Two control subjects and two COVID-19 subjects were included. In each subject a lung biopsy was taken, and five electron micrographs × two fields were analyzed, and immunolabeling was performed as previously described by our group [8]. The primary G6PDH antibody (AB 87,230 ABCAM) and a secondary goat-anti-rabbit gold 15 nm (cat: 25,112 Electron Microscopy Sciences) were employed. In panels (**A**,**B**), the arrows indicate the presence of the immune colloidal 15 nm gold marker for G6PDH antibody. The images were taken at 50,000× with a Jeol JEM-1011 electron microscope (JEOL Ltd., Tokyo, Japan) to 60 kilovolts equipped with AMT 542.391 analysis software. The histogram represents the densitometry analysis of the gold particle count; the statistical analysis was performed with The GraphPad-Prism 6 Software. Inc. (San Diego, CA, USA), 1992–2012, which was used to generate the analysis and graph. Statistical significance was determined by the Mann–Whitney rank sum test followed by the normality test (Shapiro–Wilk). Difference was considered statistically significant when *p* ≤ 0.05.

In addition, there was an induction of the expression of 15 genes in autopsies from lungs obtained from obese patients with COVID-19, and among them the G6PD gene was found [36]. An elevated activity of G6PD was associated with increased proliferation of lung cells and replacement of injured cells. Increased expression and activation of G6PD enhances progression of vasoconstriction in hypoxic lungs and the development of pulmonary hypertension. This increase in G6PD was related with elevated NADPH/NADP^+^ ratio [37]. In addition, preliminary results from our laboratory have shown that there was a significant increase in the amount of this enzyme in comparison with control subjects (*p* = 0.001, Figure 1) in postmortem biopsies of lungs from COVID-19 patients with or without comorbidities and metabolic syndrome. The enzyme was marked with G6PD antibody. This suggests that the increase of the G6PD in viral infections is necessary to elevate the NADPH, which is then used and depleted by the enzymatic and non-enzymatic antioxidant system in an attempt to balance the redox homeostasis altered by the viral infection.

In addition, the regulation of G6PD is conditioned by the NADPH/NADP ratio, which is activated after cell exposure to various extracellular oxidants that lead to a decrease in NADPH concentration. It is therefore considered that G6PD has an antioxidant effect by controlling the production of ROS. The overexpression of G6PD reduces the excess of ROS in endothelial cells treated with H_2_O_2_ plus TNFα [38].

Dehydroepiandrosterone, which is potent inhibitor of G6PD, has special relevance in the COVID-19 pandemic. Reduction of the activity of G6PD facilitates infection of human cells by the coronavirus. Uncompetitive inhibition of G6PD by oral dehydroepiandrosterone in endothelial cells leads to the depletion of intracellular NADPH, and the loss of NADPH depletes BH_4_, uncouples eNOS, and forms NO, highly reactive peroxide, and peroxynitrate [39].

### 3.2. Effects of Decreased Activity of G6PD

G6PD deficiency affects and estimated of 350–400 million people worldwide. This condition is one of the most common enzyme deficiency-related diseases worldwide [40]. Geographically, this deficiency affects individuals in Africa, European Mediterranean countries, Latin America, and Southeast and South Asia [41]. Its prevalence ranges between 3.8 and 5.2% in the United States and Europe, it is 8.5% in sub-Saharan Africa, and 7.2% in the Mediterranean area [42]. In the Al-Ahsa area in Saudi Arabia, the prevalence is of about 13% in females and 23% in males [43].

G6PD deficiency is an asymptomatic condition whose clinical consequences include hematological disturbances, such as neonatal jaundice and hemolytic anemia. It is mostly undetected until triggered by hemolytic agents such as fava bean ingestion, intake of drugs with intracellular oxidizing action, exposure to agents with intracellular oxidizing action, or exposure to bacterial and viral infections [44]. This deficiency affects erythrocytes and nucleated cells. Thus, human G6PD-deficient neutrophils display an impaired NO, O_2_^−^, and H_2_O_2_ production, which could explain their defective bactericidal effect. Unfortunately, this enzyme deficiency is further decreased with age increasing of the morbidity rate [45]. In this sense, the severity and mortality risk of SARS-CoV-2 infection has been linked with the aging process [45].

G6PD variants are categorized in five classes based on the enzymatic activity and the clinical presentation [46]. Class I variants are rare, and subjects show less than 10% of normal G6PD activity in erythrocytes. Patients usually have chronic no spherocytic hemolytic anemia [40]. Class II variants are frequently found in Mediterranean and Asian countries. This class has no more than 10% of the normal activity of G6PD in erythrocytes. Class II variants are not associated with chronic no spherocytic hemolytic anemia. Patients present acute hemolysis due to infection and food exposure (fava bean), chemicals (naphthalene mothballs), and certain drugs (antibiotics and antimalarial drugs) [47]. In these subjects, there is extensive intravascular hemolysis resulting in tubular necrosis and acute kidney failure. Class III variants are mainly found in Asian countries and Mediterranean and in American Africans. Subjects having this variant of the diseases have a moderate deficiency and show a G6PD activity of 10–60% of the normal in their erythrocytes. They have the G6PD A^−^ variant. African American males (12.2%) and females (4.1%), along with Asian males (4.3%), have the highest rates of G6PD deficiency and individuals with class III variants, and these individuals have intermittent hemolysis caused by oxidant exposure and infection [47]. Subjects with class IV variants have more than 60% of normal G6PD activity in their erythrocytes and show moderate pathological manifestations. Individuals with class V variants display higher activity of the G6PD in their erythrocytes compared to normal subjects.

The deficiency of G6PD reduces the amount of NADPH, and the activity of the antioxidant systems, which depend on the reducing equivalents produced by this enzyme. For example, a deficiency in the activity of G6PD in endothelial cells resulted in a decreased expression of eNOS, NO levels, and GSH, which leads to an increase of OS. This impaired endothelial and monocyte function elevates inflammatory cytokines, such as monocyte chemo attractant protein-1 and TNF-α [48]. The increase in TNF-α inhibited COX-2 in lung epithelial cells. It also increased the susceptibility to infection by coronavirus through a decreased phosphorylation of MAPK and NF-κB levels [49]. In macrophages, the deficiency of G6PD altered polarization contributing to overexpression of inflammatory cytokines [50]. In human granulocytes, G6PD deficiency abolished the NO production induced by LPS [51]. This deficiency also caused irreversible oxidative damage and cell death. Deficiency is more evident in erythrocytes [52] where it results in hemolytic anemia, tissue damage, and insufficient oxygen transportation when exposed to OS or to other stimuli such as a viral infection [53]. This is important because unrecognized G6PD deficiency in patients infected by SARS-CoV-2 could be associated with severe infection with worse results by causing a hemolytic crisis [54].

Oxidizing drugs such as hydroxychloroquine (HCQ) may induce methemoglobinemia and severe hemolysis in patients with G6PD deficiency [55,56]. The first case of severe hemolytic crisis was found in a seriously ill COVID-19 patient with G6PD deficiency following treatment with high doses of HCQ [57]. Several other cases associated with SARS-CoV-2 infection have subsequently been reported by other authors in people of African descent and Asians. Patients with G6PD deficiency also develop vascular endothelial dysfunction and hemolysis following initiation of HCQ treatment [58]. Chloroquine/HCQ (the most used antimalarial drug) has been proposed as a potential treatment for COVID-19 [59] because this combination inhibited SARS-CoV-2 replication in vitro. The benefits of this therapy strongly depend on the age of the patient, the clinical presentation, and the stage of the COVID-19 disease. However, it is noteworthy that the use of these drugs is contraindicated in some conditions, particularly in G6PD deficiency [55]. When G6PD deficiency has not been diagnosed, the administration of HCQ for treatment of COVID-19 results in worse outcomes associated with hemolytic crisis [60]. Development of autoantibodies seems to be responsible for the hemolytic anemia associated with COVID-19 infection [54,61]. Since HCQ therapy can induce hemolytic crises in patients with underlying G6PD deficiency or hemoglobinopathies, it should be avoided or closely monitored [61,62]. However, a recent experimental study in a murine model of G6PD deficiency suggested that high doses of HCQ for a short period of time do not lead to methemoglobinemia or clinically significant hemolytic anemia or organ damage. Moreover, there was no hemolysis in patients with G6PD deficiency exposed to low doses of hydroxychloroquine [62]. In addition, a report from a man deficient in G6PD with a severe case of COVID-19 infection treated with HCQ and intravenous n-acetylcysteine showed that n-acetylcysteine blocked the elevation of liver enzymes, hemolysis, ferritin, and C-reactive protein, allowing for the removal from a respirator and veno-venous extracorporeal membrane oxygenator and for full recovery [63]. Another study reported that α-lipoic acid attenuates the vulnerability of G6PD-deficient cells and proposed it as a treatment option for infection SARS-CoV-2 in patients with this deficiency [44].

On the other hand, there is an increased risk for cardiovascular disease, high systolic blood pressure, fibrosis, autoimmune diseases, infections, and metabolic disorders in subjects with G6PD deficiency [40]. Under stressful situations, G6PD-deficient cells cannot regenerate enough NADPH, which exacerbates GSH failure and OS [64]. Furthermore, lack of G6PD promotes cytopathic effects and viral replication. Moreover, G6PD activity determines the anti-viral response mediated by the NMRAL1 and the NF-κB pathway [41]. In G6PD-deficient human lung fibroblasts and epithelial cells infected by coronavirus 229E or enterovirus, the knockdown of NMRAL1 activates NF-κB and induces downstream antiviral gene expression, including the expression of TNF-α and the myxovirus protein 1 (MX1) promoter. It also downregulates NMRAL1. These alterations decrease viral gene expression. In contrast, the upregulation of NMRAL1 increases viral replication [1,41]. Different studies have shown that G6PD deficiency decreases the cellular immune response and is associated with an increased production of the pro-inflammatory cytokines and activation of the inflammasome [52]. For example, in a cohort of 182 patients with SARS-CoV-2, there was a higher frequency of G6PD enzyme deficiency in patients with severe symptoms [65]. Human fibroblasts deficient in G6PD and lung epithelial carcinoma A549 cells treated with interference G6PD-RNA in vitro showed a decreased viability and an elevation of viral replication [66]. Another study showed that there is an increase in the expression of the TNF-α in human G6PD-deficient alveolar epithelial cells with carcinoma after the infection with HCoV-229E. This was also observed in G6PD-deficient peripheral blood mononuclear cells. These monocytes cells showed impaired inflammasome activation [1,67]. Fibroblasts and lung epithelial cells with G6PD deficiency were more susceptible to coronavirus 229E infection due to increased production of ROS and depletion of the GSH [1]. This suggests that G6PD deficiency allows for viral proliferation and impairs the cellular immune response including neutrophil extracellular trap formation. This is due to an unbalanced redox homeostasis, downregulation of interleukin 1β expression, and increased inflammation through upregulated NF-κB-mediated pro-inflammatory cytokines [68].

The G6PD deficiency also impairs the activation of the inflammasome due to reduced ROS production via NADH-oxidase, and it therefore reduces the antiviral response [69]. The inflammasome activation causes an increase in the cytokine storm in patients infected by SARS-CoV-2 [70]. This suggests that G6PD is required for the maintenance of the innate immune response, the inflammasome activation, and pathogen clearance through redox homeostasis [69]. Moreover, the altered or deficient activity of G6PD is related to different pathologies such as insulin resistance, diabetes, anemia, hypertension, autophagy, infection, and inflammation [36]. In addition, glycosylation of proteins and hyperglycemia is higher in diabetes and oxidative stress, causing a decrease in the activity of G6PD and its protective mechanisms, particularly in patients with inherited G6PD deficiency. Deficiency of G6PD also polarizes the expression of inflammatory cytokines in human monocyte and macrophages that support of adaptive immunity. A pro-inflammatory phenotype of monocytes is enhanced by diabetes and hyperglycemia inducing chronic inflammation. There is also an insufficient M2 profibrotic TGF-β signaling similar to that present in G6PD-deficient subjects. Moreover, deficiency of G6PD or GSH elevates inflammation and respiratory distress. These conditions are common in several diseases, including diabetes, chronic obstructive pulmonary disease, and several viral infections [41]. Studies should also aim to determine the role of G6PD in nucleated cells since they play a role in regulating cell proliferation, cell death, autophagy, inflammation, and tumorigenesis. At present, most studies have been centered on the effect of this enzyme on erythrocytes. G6PD deficiency also reduces replicative potential in human fibroblasts, leading to an early onset of senescence [66].

## 4. Conclusions

**Figure 2:**
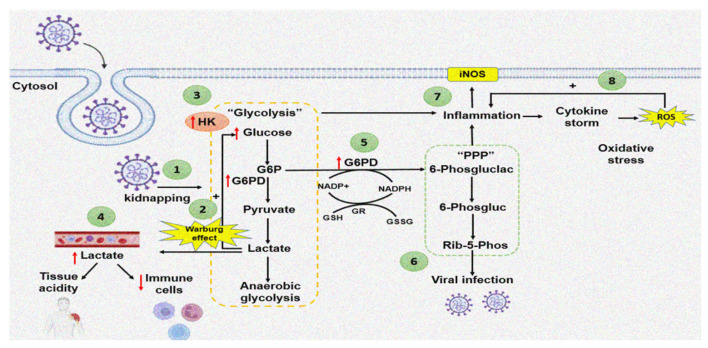
Effect of SARS-CoV-2 infection on G6PD activity. The red arrow up represents increase and down decrease (1) COVID-19 entry and cell sequestration. (2) Increase in lactate stimulates increase in glucose: “Warburg effect”. (3) Increased HK associated with the Warburg effect. (4) Lactate accumulates in the bloodstream, which promotes tissue acidification and depletion of immune system cells. (5) The increase in G6PD provides reducing equivalents for different antioxidant enzymes. (6) Nucleic acids generated by the PPP are used for viral infection. (7) The inflammation stimulates iNOS. (8) Positive feedback between inflammation-cytokine storm-ROS.

An increase in the activity of G6PD raises NADPH, which is used by the enzymatic and non-enzymatic antioxidant systems to counteract the OS caused by the cytokine storm in COVID-19 patients. However, in subjects with G6PD deficiency, the amount of NADPH is reduced, rendering these patients more susceptible to viral infection. G6PD deficiency is associated to loss of the redox homeostasis, which could result in severe pneumonia and a fatal outcome. Figure 2 summarizes the way by which the SARS-CoV-2 virus may increase the G6PD activity through of the Warburg effect.

## Data Availability

The datasets generated and analyzed during the current study are available from the corresponding author on reasonable request.

## Abbreviations

G-6-P	glucose 6 phosphate
G6PD	Glucose-6-phosphate dehydrogenase
GR	glutathione reductase
GSSG	oxidized glutathione
GSH	glutathione
HK	hexokinase
iNOS	inducible nitric oxide synthase
NADPH	nicotinamide adenine dinucleotide phosphate
PPP	pentose phosphate pathway
ROS	reactive oxygen species
COVID-19	Coronavirus disease 2019
SARS-CoV-2	Severe acute respiratory syndrome
G6PD	Glucose-6-phosphate dehydrogenase
NADPH	Nicotinamide-adenine dinucleotide phosphate
GHS	glutathione
PPP	pentose phosphate pathway
ATP	adenosine-5-triphosphate
HK	hexokinase
G6P	glucose-6-phosphat
iNOS	inducible nitric oxide
eNOS	endothelial nitric oxide synthase
OS	oxidative stress
TNF-α	tumor necrosis factor alpha
COX-2	cyclooxygenase
MAPK	mitogen-activated protein kinases
NF-κB	nuclear factor κB
NO	nitric oxide
LPS	lipopolysaccharide
NMRAL-1	NmrA-like family domain-containing protein 1
MX1	Myxovirus protein 1

## References

[1] Wu Y.H., Tseng C.P., Cheng M.L., Ho H.Y., Shih S.R., Chiu D.T. (2008). Glucose-6-phosphate dehydrogenase deficiency enhances human coronavirus 229E infection. J. Infect. Dis..

[2] Buinitskaya Y., Gurinovich R., Wlodaver C.G., Kastsiuchenka S. (2020). Centrality of G6PD in COVID-19: The biochemical rationale and clinical implications. Front. Med..

[3] Wu S., Yu L., Fu X., Yan X., Lin Q., Liu L., Liang H., Li N. (2018). iTRAQ-based proteomic profile analysis of ISKNV-infected CPB cells with emphasizing on glucose metabolism, apoptosis and autophagy pathways. Fish Shellfish Immunol..

[4] Aydemir D., Nuray U.N. (2020). Is glucose-6-phosphate dehydrogenase enzyme deficiency a factor in Coronavirus-19 (COVID-19) infections and deaths?. Pathog. Glob. Health.

[5] Bonilla J.F., Sánchez M.C., Chuaire L. (2007). Glucosa-6-fosfato deshidrogenasa (G6PD): Respuesta de los hematíes y otras células humanas a la disminución en su actividad. Colomb. Med..

[6] Kumar S.S., Tarikul I.M., Eckhoff G., Amir H.M., Kashfi Q.S., Muraduzzaman A.K.M., Sarower B.G., Shahidullah M., Abdul M.M., Tahura S. (2016). Molecular analysis of glucose-6-phosphate dehydrogenase gene mutations in Bangladeshi individuals. PLoS ONE.

[7] Peiró C., Romacho T., Azcutia V., Villalobos L., Fernández E., Bolaños J.P., Moncada S., Sánchez-Ferrer C.F. (2016). Inflammation, glucose, and vascular cell damage: The role of the pentose phosphate pathway. Cardiovasc. Diabetol..

[8] Soria-Castro E., Soto M.E., Guarner-Lans V., Rojas G., Perezpeña-Diazconti M., Críales-Vera S.A., Manzano-Pech L., Pérez-Torres I. (2021). The kidnapping of mitochondrial function associated with the SARS-CoV-2 infection. Histol. Histopathol..

[9] Barberis E., Timo S., Amede E., Vanella V.V., Puricelli C., Cappellano G., Raineri D., Cittone M.G., Rizzi E., Pedrinelli A.R. (2020). Large-scale plasma analysis revealed new mechanisms and molecules associated with the host response to SARS-CoV-2. Int. J. Mol. Sci..

[10] Fontaine K.A., Sanchez E.L., Camarda R., Lagunoff M. (2015). Dengue virus induces and requires glycolysis for optimal replication. J. Virol..

[11] Soto M.E., Guarner-Lans V., Díaz-Díaz E., Manzano-Pech L., Palacios-Chavarría A., Valdez-Vázquez R.R., Aisa-Álvarez A., Saucedo-Orozco H., Pérez-Torres I. (2022). Hyperglycemia and loss of redox homeostasis in COVID-19 patients. Cells.

[12] Ceriello A., Prattichizzo F. (2021). Pharmacological management of COVID-19 in type 2 diabetes. J. Diabetes Complicat..

[13] De Candia P., Nicolucci A., Ceriello A. (2022). Elevated HbA1c levels in pre-Covid-19 infection increases the risk of mortality: A sistematic review and meta-analysis. Diabetes Metab. Res. Rev..

[14] Meloni L., Manca M.R., Loddo I., Cioglia G., Cocco P., Schwartz A., Muntoni S., Muntoni S. (2008). Glucose-6-phosphate dehydrogenase deficiency protects against coronary heart disease. J. Inherit. Metab. Dis..

[15] Vander H.M.G., Cantley L.C., Thompson C.B. (2009). Understanding the Warburg effect: The metabolic requirements of cell proliferation. Science.

[16] Icard P., Lincet H., Wu Z., Coquerel A., Forgez P., Alifano M., Fournel L. (2021). The key role of Warburg effect in SARS-CoV-2 replication and associated inflammatory response. Biochimie.

[17] Yu Y., Maguire T.G., Alwine C.J. (2011). Human cytomegalovirus activates glucose transporter 4 expression to increase glucose uptake during infection. J. Virol..

[18] Singh S., Singh P.K., Suhail H., Arumugaswami V., Pellett P.E., Giri S., Kumar A. (2020). AMP-activated protein kinase restricts zika virus replication in endothelial cells by potentiating innate antiviral responses and inhibiting glycolysis. J. Immunol..

[19] Sen S., Kaminiski R., Deshmane S., Langford D., Khalili K., Amini S., Datta P.K. (2015). Role of hexokinase-1 in the survival of HIV-1-infected macrophages. Cell Cycle.

[20] Ramière C., Rodriguez J., Enache L.S., Lotteau V., André P., Diaz O. (2014). Activity of hexokinase is increased by its interaction with hepatitis C virus protein NS5A. J. Virol..

[21] Julin K., Johansen L.H., Sommer A.I. (2009). Infectious pancreatic necrosis virus (IPNV) IPNV infection can alter the regulation of transcription of G6PDH. J. Virol. Methods.

[22] Godoy-Lugo J.A., Miranda-Cruz M.M., Rosas-Rodríguez J.A., Adan-Bante N.P., Icedo-García R.G., Soñanez-Organis J. (2019). Hypoxia inducible factor-1 regulates WSSV-induced glycolytic genes in the white shrimp Litopenaeusvannamei. Fish Shellfish Immunol..

[23] Salvemini F., Franzé A., Iervolino A., Filosa S., Salzano S., Ursini M.V. (1999). Enhanced glutathione levels and oxide resistance mediated by increased glucose-6-phosphate dehydrogenase expression. J. Biol. Chem..

[24] Chen I.T., Aoki T., Huang Y.T., Hirono I., Chen T.C., Huang J.Y. (2011). White spot Syndrome virus induces metabolic changes resembling the Warburg effect in shrimp hemocytes in the early stage of infection. J. Virol..

[25] Ritter J.B., Wahl A.S., Freund S., Genzel Y., Reichl U. (2010). Metabolic effects of influenza virus infection in cultured animal cells: Intra- and extracellular metabolite profiling. BMC Syst. Biol..

[26] Li Y.P., Handberg K.J., Juul-Madsen H.R., Zhang M.F., Jørgensen P.H. (2007). Transcriptional profiles of chicken embryo cell cultures following infection with infectious bursal disease virus. Arch. Virol..

[27] Galván C.A., Elbarcha O.C., Fernández E.J., Beltramo D.M., Soria N.W. (2011). Development of a method to control the RNA extraction and reverse transcription steps for the detection of dengue virus present in human blood samples. Genet. Test. Mol. Biomark..

[28] Turner M., Dawson P.J. (1970). Enzyme, and isoenzyme alterations in Friend disease. Br. J. Cancer.

[29] Clemente-Moreno M.J., Díaz-Vivancos P., Barba-Espín G., Hernández J.A. (2010). Benzothiadiazole and l-2-oxothiazolidine-4-carboxylic acid reduce the severity of Sharka symptoms in pea leaves: Effect on antioxidative metabolism at the subcellular level. Plant Biol..

[30] Diamond D.L., Syder A.J., Jacobs J.M., Sorensen C.M., Walters K.A., Proll S.C., McDermott J.E., Gritsenko M.A., Zhang Q., Zhao R. (2010). Temporal proteome and lipidome profiles reveal hepatitis C virus-associated reprogramming of hepatocellular metabolism and bioenergetics. PLoS Pathog..

[31] Nadeem A., Al-Harbi N., Ahmad S., Ibrahim K., Siddiqui N., Al-Harbi M. (2018). Glucose-6-phosphate dehydrogenase inhibition attenuates acute lung injury through reduction in NADPH oxidase-derived reactive oxygen species. Clin. Exp. Immunol..

[32] Doustimotlagh A.H., Eftekhari M. (2021). Glucose-6-phosphatedehydrogenase inhibitor for treatment of severe COVID-19: Polydatin. Clin. Nutr. ESPEN.

[33] Liu B., Fang M., He Z., Cui D., Jia S., Lin X., Xu X., Zhou T., Liu W. (2015). Hepatitis B virus stimulates G6PD expression through HBx-mediated Nrf2 activation. Cell Death Dis..

[34] Hu T., Li Y.S., Chen B., Chang Y.F., Liu G.C., Hong Y., Chen H.L., Xiyang Y.B. (2015). Elevated glucose-6-phosphate dehydrogenase expression in the cervical cancer cases is associated with the cancerigenic event of high-risk human papillomaviruses. Exp. Biol. Med..

[35] Bouchla A., Kriebardis A.G., Georgatzakou H.T., Fortis S.P., Thomopoulos T.P., Lekkakou L., Markakis K., Gkotzias D., Panagiotou A., Papageorgiou E.G. (2022). Red blood cell abnormalities as the mirror of SARS-CoV-2 disease severity: A pilot study. Front. Physiol..

[36] Santos e Silva J.C., Pereira V.A., Harumi Y.N.I., Noronha N.Y., Aquino R., Giddaluru J., Durão L., Costa-Martins A.G., Schuch V., Moraes-Vieira P.M. (2021). Gene signatures of autopsy lungs from obese patients with COVID-19. Clin. Nutr. ESPEN.

[37] Gupte R.S., Rawat D.K., Chettimada S., Cioffi D.L., Wolin M.S., Gerthoffer W.T. (2010). Activation of glucose-6-phosphate dehydrogenase promotes acute hypoxic pulmonary artery contraction. J. Biol. Chem..

[38] Stanton R. (2012). Glucose-6-phosphate dehydrogenase, NADPH, and cell survival. IUBMB Life.

[39] Nyce J. (2021). Alert to US physicians: DHEA, widely used as an OTC androgen supplement, may exacerbate COVID-19. Endocr. Relat. Cancer.

[40] Jain S.K., Parsanathan R., Levine S.N., Bocchini J.A., Holick M.F., Vanchiere J.A. (2020). The potential link between inherited G6PD deficiency, oxidative stress, and vitamin D deficiency and the racial inequities in mortality associated with COVID-19. Free Radic. Biol. Med..

[41] Yang H.C., Ma T.H., Tjong W.Y., Stern A., Chiu D.T.Y. (2021). G6PD deficiency, redox homeostasis, and viral infections: Implications for SARS-CoV-2 (COVID-19). Free Radic. Res..

[42] Khneisser I., Farra C. (2021). Chloroquine and the potential adverse outcome in undiagnosed G6PD-deficient cases infected with COVID-19. J. Med. Screen..

[43] Al-Abdi S., Al-Aamri M. (2021). G6PD deficiency in the COVID-19 pandemic: Ghost within Ghost. Hematol. Oncol. Stem. Cell Ther..

[44] Sgherza N., Dalfino L., Palma A., Vitucci A., Campanale D., Grasso S., Pellegrino Musto P. (2020). Hemolysis, or not Hemolysis, that is the Question. Use of Hydroxychloroquine in a Patient with COVID-19 Infection and G6PD Deficiency. Mediterr. J. Hematol. Infect. Dis..

[45] Abdel-Hafez S.M.N. (2020). Glucose-6-phosphate dehydrogenase deficiency enhances Covid-19 infection in elderly people. Bratisl. Lek. Listy..

[46] Onori M.E., Ricciardi T.C., Urbani A., Minucci A. (2021). Glucose-6-phosphate dehydrogenase deficiency and hydroxychloroquine in the COVID-19 era: A mini review. Mol. Biol. Rep..

[47] Garcia A.A., Koperniku A., Ferreira J.C.B., Mochly-Rosen D. (2021). Treatment strategies for glucose-6-phosphate dehydrogenase deficiency: Past and future perspectives. Trends Pharmacol. Sci..

[48] Parsanathan R., Jain S.K. (2019). Glucose-6-phosphate dehydrogenase deficiency increases cell adhesion molecules and activates human monocyte-endothelial cell adhesion: Protective role of l-cysteine. Arch. Biochem. Biophys..

[49] Yang H.C., Cheng M.L., Ho H.Y., Chiu D.T. (2011). The microbicidal and cytoregulatory roles of NADPH oxidases. Microbes. Infect..

[50] Parsanathan R., Jain S.K. (2020). Glucose-6-phosphate dehydrogenase (G6PD) deficiency is linked with cardiovascular disease. Hypertens. Res..

[51] Youssef J.G., Zahiruddin F., Youssef G., Padmanabhan S., Ensor J., Pingali S.R., Zu Y., Sahay S., Iyer S.P. (2021). G6PDdeficiency and severity of COVID19 pneumonia and acute respiratory distress syndrome: Tip of the iceberg?. Ann. Hematol..

[52] Chinevere T.D., Murray C.K., Grant E., Johnson G.A., Duelm F., Hospenthal D.R. (2006). Prevalence of glucose-6-phosphate dehydrogenase deficiency in U.S. Army personnel. Mil. Med..

[53] Sasi S., Yassin M.A., Nair A.P., Al Maslamani M.S. (2020). A Case of COVID-19 in a Patient with Asymptomatic Hemoglobin D Thalassemia and Glucose-6-Phosphate Dehydrogenase Deficiency. Am. J. Case Rep..

[54] Brenda D., Jamerson T., Haryadi H., Bohannon A. (2020). Glucose-6-phosphate dehydrogenase deficiency: An actionable risk factor for patients with COVID-19?. Arch. Med. Res..

[55] Laslett N., Hibbs J., Hallett M., Ghaneie A., Zemba-Palko V. (2021). Glucose-6-hosphate dehydrogenase deficiency-associated hemolytic anemia and methemoglobinemia in a patient treated with hydroxychloroquine in the era of COVID-19. Cureus.

[56] Scholkmann F., Restin T., Ferrari M., Quaresima V. (2021). The Role of methemoglobin and carboxyhemoglobin in COVID-19: A Review. J. Clin. Med..

[57] Vick D.J. (2021). Evaluation of glucose-6-phosphate dehydrogenase (G6PD) status in US military and VA patients with COVID-19 infection. BMJ. Mil. Health.

[58] Da Rocha J.E.B., Othman H., Tiemessen C.T., Botha G., Ramsay M., Masimirembwa C., Adebamowo C., Choudhury A., Brandenburg J.T., Matshaba M. (2021). *G6PD* distribution in sub-Saharan Africa and potential risks of using chloroquine/hydroxychloroquine based treatments for COVID-19. Pharm. J..

[59] Capoluongo E.D., Amato F., Castaldo G. (2020). The friendly use of chloroquine in the COVID-19 disease: A warning for the G6PD-deficient males and for the unaware carriers of pathogenic alterations of the G6PD gene. Clin. Chem. Lab. Med..

[60] AbouYabis A.N., Thompson G. (2021). Hemolytic anemia complicating COVID-19 infection. J. Hematol..

[61] Mastroianni F., Colombie V., Claes G., Gilles A., Vandergheynst F., Place S. (2020). Hydroxychloroquine in a G6PD-deficient patient with COVID-19 complicated by haemolytic anaemia: Culprit or innocent bystander?. Eur. J. Case Rep. Intern. Med..

[62] Kumar N., AbdulRahman A., AlAwadhi A.I., AlQahtani M. (2021). Is glucose-6-phosphatase dehydrogenase deficiency associated with severe outcomes in hospitalized COVID-19 patients?. Sci. Rep..

[63] Ibrahim H., Perl A., Smith D., Lewis T., Kon Z., Goldenberg R., Yarta K., Staniloae C., Williams M. (2020). Therapeutic blockade of inflammation in severe COVID-19 infection with intravenous N-acetylcysteine. Clin. Immunol..

[64] Ho H.Y., Cheng M.L., Weng S.F., Chang L., Yeh T.T., Shih S.R., Chiu D.T. (2008). Glucose-6-phosphate dehydrogenase deficiency enhances enterovirus infection. J. Gen. Virol..

[65] Littera R., Campagna M., Deidda S., Angioni G., Cipri S., Melis M., Firinu D., Santus S., Lai A., Porcella R. (2020). Human Leukocyte antigen complex and other immunogenetic and clinical factors influence susceptibility or protection to SARS-CoV-2 infection and severity of the disease course. The Sardinian experience. Front. Immunol..

[66] Yi-Hsuan W., Tsun-Yee C.D., Hsin-Ru L., Hsiang-Yu T., Mei-Ling C., Hung-Yao H. (2015). Glucose-6-phosphate dehydrogenase enhances antiviral response through downregulation of NADPH sensor HSCARG and upregulation of NF-κB signaling. Viruses.

[67] Nabavi S.F., Habtemariam S., Sureda A., Banach M., Berindan-Neagoe I., Cosmin A.C., Mahdi B., Mohammad S.B., Seyed M.N. (2020). Glucose-6-phosphate dehydrogenase deficiency and SARS-CoV-2 mortality: Is there a link and what should we do?. Clin. Biochem..

[68] Elhabyan A., Elyaacoub S., Sanad E., Abukhadra A., Elhabyan A., Dinu V. (2020). The role of host genetics in susceptibility to severe viral infections in humans and insights into host genetics of severe COVID-19: A systematic review. Virus Res..

[69] Ratajczak M.Z., Kucia M. (2020). ARS-CoV-2 infection and overactivation of Nlrp3 inflammasome as a trigger of cytokine “storm” and risk factor for damage of hematopoietic stem cells. Leukemia.

[70] Yen W.C., Wu Y.H., Wu C.C., Lin H.R., Stern A., Chen S.H., Shu J.C., Tsun-Yee C.D. (2020). Impaired inflammasome activation and bacterial clearance in G6PD deficiency due to defective NOX/p38 MAPK/AP-1 redox signaling. Redox. Biol..

